# Eplerenone nanocrystals engineered by controlled crystallization for enhanced oral bioavailability

**DOI:** 10.1080/10717544.2021.2008051

**Published:** 2021-11-29

**Authors:** Muhammad Ayub Khan, Muhammad Mohsin Ansari, Sadia Tabassam Arif, Abida Raza, Ho-Ik Choi, Chang-Wan Lim, Ha-Yeon Noh, Jin-Su Noh, Salman Akram, Hafiz Awais Nawaz, Muhammad Ammad, Abir Abdullah Alamro, Amani Ahmed Alghamdi, Jin-Ki Kim, Alam Zeb

**Affiliations:** a Riphah Institute of Pharmaceutical Sciences, Riphah International University, Islamabad, Pakistan; b Nanomedicine Research Laboratory, National Institute of Lasers and Optronics (NILOP), PIEAS, Islamabad, Pakistan; c College of Pharmacy, Institute of Pharmaceutical Science and Technology, Hanyang University, Ansan, Republic of Korea; d Laboratory for the Study of Rheology and the Adhesion of Medical Adhesives, IPREM, University of Pau and Pays de l'Adour, Pau, France; e Institute of Pharmaceutical Sciences, University of Veterinary and Animal Sciences, Lahore, Pakistan; f Drug Testing Laboratory, Lahore, Pakistan; g Department of Biochemistry, College of Science, King Saud University, Riyadh, Saudi Arabia

**Keywords:** Eplerenone, poorly aqueous solubility, nanocrystals, controlled crystallization, dissolution rate and bioavailability, acute toxicity study

## Abstract

Poor aqueous solubility of eplerenone (EPL) is a major obstacle to achieve sufficient bioavailability after oral administration. In this study, we aimed to develop and evaluate eplerenone nanocrystals (EPL-NCs) for solubility and dissolution enhancement. D-optimal combined mixture process using Design-Expert software was employed to generate different combinations for optimization. EPL-NCs were prepared by a bottom-up, controlled crystallization technique during freeze-drying. The optimized EPL-NCs were evaluated for their size, morphology, thermal behavior, crystalline structure, saturation solubility, dissolution profile, *in vivo* pharmacokinetics, and acute toxicity. The optimized EPL-NCs showed mean particle size of 46.8 nm. Scanning electron microscopy revealed the formation of elongated parallelepiped shaped NCs. DSC and PXRD analysis confirmed the crystalline structure and the absence of any polymorphic transition in EPL-NCs. Furthermore, EPL-NCs demonstrated a 17-fold prompt increase in the saturation solubility of EPL (8.96 vs. 155.85 µg/mL). The dissolution rate was also significantly higher as indicated by ∼95% dissolution from EPL-NCs in 10 min compared to only 29% from EPL powder. EPL-NCs improved the oral bioavailability as indicated by higher AUC, *C*_max_, and lower *T*_max_ than EPL powder. Acute oral toxicity study showed that EPL-NCs do not pose any toxicity concern to the blood and vital organs. Consequently, NCs prepared by controlled crystallization technique present a promising strategy to improve solubility profile, dissolution velocity and bioavailability of poorly water-soluble drugs.

## 1. Introduction

The growing number of poorly water soluble drugs remains a huge challenge in the development, optimization, and formulation of new drug molecules. It is estimated that approximately 90% of newly discovered drugs possess low or pH-dependent aqueous solubility (Fontana et al., 2018). Biopharmaceutics Classification System (BCS) suggests that nearly 70% of these compounds belong to class II whereas 20% fall in class IV (Peltonen & Hirvonen, 2018). The therapeutic effectiveness of such drugs is reduced as they encounter poor oral bioavailability because of low dissolution and limited absorption from the gastrointestinal (GI) membrane (Shegokar & Müller, 2010). Their solubilizing capacity and consequently the oral bioavailability can be enhanced by different approaches including their solid-state modification to amorphous forms (Williams et al., 2013; Hashim Ali et al., 2019; Kim et al., 2021). However, drugs in an amorphous state have reduced stability and the possibility of reconversion to crystalline form during processing and storage also exists. This transition from amorphous to the crystalline state is undesirable and alters the dissolution rate of drug molecules (Hancock & Parks, 2000). Alternatively, drug nanocrystals (NCs) having particle size down to nanometer range can be utilized to improve their dissolution and solubility profiles while avoiding the problems associated with the unwanted reconversion (Fontana et al., 2018).

NCs are carrier-free, crystalline drug nanoparticles with a size typically in the range of 10–800 nm (Gigliobianco et al., 2018). NCs present a versatile platform for drug delivery applications that offer carrier-free systems, higher drug-loading (theoretically up to 100%), and superior colloidal stability compared to their nanoparticulate counterparts such as liposomes, lipid- and polymer-based nanoparticles, and polymeric micelles (Chen et al., 2020). The small particle size and large surface area of drug NCs enhance their solubility, dissolution rates, adhesiveness, and residence time with GI mucosa thereby improving their oral bioavailability. Some other advantages are the opportunity of incorporating NCs into different dosage forms and their feasibility for administration via oral, parenteral, topical, ocular, and pulmonary routes (Schroeter et al., 2010; Zhang et al., 2011; Kakran et al., 2012; Chen et al., 2014; Lu et al., 2016; Gigliobianco et al., 2018). The novel preparation techniques and better control of properties have led to the production of NCs with enhanced functional characteristics and extended *in vivo* applications such as prolonged blood circulation, targeted delivery, and drug tracking applications (Liu et al., 2019). NCs are most commonly produced by top-down and bottom-up techniques. In the top-down technique, the bulk drug is subjected to particle size reduction by wet pearl milling, bead milling and high-pressure homogenization, whereas the bottom-up technique involves the growing of NCs from the molecular level by using evaporation and precipitation methods (Keck & Müller, 2006; Kakran et al., 2012; Peltonen & Hirvonen, 2018; Chen et al., 2020). Among the bottom-up approaches, solvent–antisolvent precipitation is a widely used method for improving the solubility and dissolution profiles of poorly soluble drugs. The use of stabilizers (surfactants), higher operational time and energy input, lower yield and non-uniform particle size distribution are some of the factors that render top-down technique less favorable. On the other hand, bottom-up approaches are relatively simple and economical, require lesser energy input and can be scaled up effectively. The use of controlled crystallization further enhances the efficiency of bottom-up technique as no surfactant is required to prevent crystal growth and the residual solvents can also be removed during freeze drying process (Kipp, 2004; Koradia et al., 2018).

Eplerenone (EPL) is an aldosterone receptor antagonist and is utilized to manage hypertension and chronic heart failure (Seferovic et al., 2015). Like other BCS class II drugs, EPL also possesses low aqueous solubility and dissolution-dependent absorption with the resultant low oral bioavailability (Ozdemir et al., 2018). Therefore, its solubilizing capability needs to be enhanced for achieving efficient absorption and sufficient bioavailability. The current work is intended to develop and optimize eplerenone nanocrystals (EPL-NCs) by using a D-optimal mixture design process. EPL-NCs were prepared by using a novel bottom-up, controlled crystallization technique during freeze-drying, and the optimized formulation was subsequently studied for different physicochemical properties. The *in vitro* saturation solubility and dissolution profile of EPL-NCs was measured in 0.1 N HCl solution as a gastric fluid. The *in vivo* pharmacokinetic parameters were determined after oral administration of EPL-NCs to rats. Furthermore, a single-dose acute oral toxicity study on the developed EPL-NCs was conducted in mice for 14 days. The graphical summary of our study is presented in Supplementary Fig. 1.

## 2. Materials and methods

### 2.1. Materials

Eplerenone was a generous gift from Highnoon Laboratories Ltd. (Lahore, Pakistan). Tertiary butyl alcohol was purchased from Daejung Chemicals Co., Ltd. (Siheung, Republic of Korea). Mannitol was procured from Sigma-Aldrich (St. Louis, MO). All other chemicals used in this study were of analytical grade.

### 2.2. Experimental design for optimization of EPL-NCs

The process and composition of EPL-NCs were optimized by using Design-Expert software (version 12, Stat-Ease Inc., Minneapolis, MN). D-optimal combined mixture process design was applied to generate different runs of formulations. For this purpose, solvent (tertiary butyl alcohol) to antisolvent (water) ratio (total volume = 10 mL), cryoprotectant (mannitol) concentration, and freezing method (slow or rapid) were chosen as independent variables. On the other hand, NCs size and dissolution efficiency were selected as dependent variables. These variables along with their levels and constraints are presented in Table 1. EPL concentration was kept constant at 200 mg in all formulations. The software generated runs of formulations with different composition and process variables were practically prepared and their responses were measured in terms of NCs size and dissolution efficiency.

**Table 1.** Variables and their levels and constraints in D-optimal combined mixture process design.

	Independent variables	Lower level	Upper level
Mixture components (*X*=*A*+*B*)	*A*=solvent (mL)	1	9
	*B*=antisolvent (mL)	1	9
Process factors	*C*=cryoprotectant concentration (%)	3	15
	*D*=freezing method	Rapid	Slow
	Dependent variables	Constraints	
Response 1	Nanocrystal size (nm)	Minimum	
Response 2	Dissolution efficiency (%)	Maximum	

The measured responses of both dependent variables (NCs size and dissolution efficiency) were introduced in the response column of Design-Expert software (Stat-Ease Inc., Minneapolis, MN). These responses were fitted to empirical model which interpreted the behavior of independent variables. Statistical analysis of the experimental data was carried to check the adequacy of proposed model by applying ANOVA test with Design-Expert software (Stat-Ease Inc., Minneapolis, MN).

### 2.3. Preparation of EPL-NCs

EPL-NCs were prepared by utilizing a controlled crystallization technique during freeze-drying as previously reported (Van Drooge et al., 2004; De Waard et al., 2008). Concisely, EPL was dissolved in tertiary butyl alcohol whereas mannitol solution was prepared in distilled water. EPL and mannitol solutions were separately heated at 60 °C in a water bath and then mixed in a freeze dryer vial. The resultant mixture was then frozen either by rapid or slow freezing method and subsequently freeze-dried by using a lyophilizer (TFD5503, IlShin BioBase Co., Ltd., Yangju, Republic of Korea). The mixtures were instantaneously cooled at −80 °C in rapid freezing, while they were slowly cooled to −25 °C at a rate of 0.5 °C/min in slow freezing method.

### 2.4. Physicochemical characterization of EPL-NCs

#### 2.4.1. Particle size, size distribution, and morphology analysis

The average size and polydispersity index (PDI) of EPL-NCs were analyzed by using dynamic light scattering technique with Zetasizer ZS 90 (Malvern Instruments, Malvern, UK). Samples were suitably diluted with deionized water (Rana et al., 2020). The morphology of optimized EPL-NCs was examined with scanning electron microscopy (SEM, VEGA 3 LMU, Tescan Analytics, Brno, Czech Republic). For this purpose, EPL-NCs formulation was spread on carbon-coated tape and a thin layer of gold was used to sputter the sample under vacuum and were subsequently imaged with SEM at an accelerating voltage of 10 kV (Zeb et al., 2017a).

#### 2.4.2. Molecular dynamics of EPL-NCs

The molecular vibrations of EPL, mannitol, their physical mixture and optimized EPL-NCs were characterized by using attenuated total reflectance Fourier transform infrared spectrophotometer (FTIR, Eco Alpha II-Bruker, Billerica, MA). The FTIR spectra of the samples were obtained between 4000 and 400 cm^−1^ at a resolution of 4 cm^−1^ (Khan et al., 2020).

#### 2.4.3. Thermal and crystalline behavior of EPL-NCs

Thermal attributes of EPL, mannitol, their physical mixture and optimized EPL-NCs were investigated by using a differential scanning calorimeter (DSC-60; Shimadzu Corporation, Kyoto, Japan). The DSC analysis was carried out by accurately weighing the sample (3–5 mg) in an aluminum pan and heating it between 50 and 300 °C at a rate of 10 °C/min. A stream of nitrogen was also used to purge the system (Kim et al., 2018; Rizvi et al., 2019).

The crystalline attributes of EPL, mannitol, their physical mixture and optimized EPL-NCs were evaluated using a powder X-ray diffractometer (D8 Advance-Bruker, Billerica, MA). The PXRD patterns of all the samples were obtained between the 2*θ* range of 10–80  at a scanning rate of 4 /min. The instrument was operated at 40 mA current and 40 kV voltage (Rubab et al., 2021).

### 2.5. Saturation solubility and *in vitro* dissolution of EPL-NCs

The saturation solubility and *in vitro* dissolution of optimized EPL-NCs were evaluated in 0.1 N HCl solution as gastric medium. For the determination of saturation solubility, an excess amount of EPL-NCs was added to 10 mL of 0.1 N HCl and the resultant dispersion was shaken at 50 rpm and 37 °C for 72 h in an orbital shaking incubator. The samples were then removed from the shaker, filtered through 0.45 μm PVDF syringe filters, suitably diluted and analyzed for drug contents by using a UV–visible spectrophotometer (UV-1800, Shimadzu Corporation, Kyoto, Japan) at a wavelength of 241 nm (Kendre & Chaudhari, 2017). The saturation solubility of EPL powder was also determined by using the same procedure.

The dissolution profile of the optimized EPL-NCs and EPL powder was determined in USP type II apparatus using 0.1 N HCl solution as a dissolution medium. Briefly, an accurately weighed sample (equivalent to 50 mg of EPL) was added to 1000 mL of dissolution medium maintained at 37 ± 0.5 °C and stirred at 50 rpm. The dissolution medium also contained 0.5% (w/v) Tween 80 to maintain the sink conditions throughout the experiments. Subsequently, samples (3 mL) were withdrawn at predefined time intervals of 10, 20, 30, 40, 60, 90, and 120 min. The apparatus was immediately replenished with an equal volume of fresh dissolution medium to keep the final volume constant. Finally, the removed samples were filtered and analyzed for EPL content with a UV–visible spectrophotometer at 241 nm.

### 2.6. Experimental animals

Male Sprague Dawley rats (300 ± 20 g) were used to assess the *in vivo* pharmacokinetic profile, whereas male Swiss albino mice (30 ± 5 g) were used to evaluate single-dose acute toxicity after oral administration of the optimized EPL-NCs. All experimental animals were acquired from the National Institute of Health (Islamabad, Pakistan). During the one-week acclimatization period before the experiments, animals were retained in cages under standard laboratory conditions (25 ± 0.5 °C, RH of 40–60%) and provided with alternative light–dark cycles and an adequate supply of food and water. All experiments involving animals were approved by the Institutional Research and Ethics Committee of Riphah Institute of Pharmaceutical Sciences (approval # REC/RIPS/2021/019). Furthermore, all animal studies were conducted according to the NIH policies and the animal welfare act.

### 2.7. *In vivo* pharmacokinetics of EPL-NCs

#### 2.7.1. Oral administration and blood sampling

Rats were randomly divided into two groups (*n* = 3) to assess the pharmacokinetics of EPL-NCs and EPL powder. Rats in their respective groups were either administered EPL-NCs or EPL powder at a dose equivalent to 10 mg/kg of EPL. For this purpose, EPL-NCs and EPL powder were dispersed in 2 mL of water (concentration equal to 5 mg/mL of EPL) and were administered orally using oral gavage. Subsequently, blood samples of 0.5 mL were taken at pre-decided time intervals of 0.25, 0.5, 1, 2, 4, 8, and 12 h from the jugular vein of rats. Blood samples were added to heparinized tubes and were instantly centrifuged at 7000 rpm for 10 min. Plasma was isolated and preserved at −80 °C till further analysis (Özdemir et al., 2018).

#### 2.7.2. Plasma processing and drug quantification with HPLC

EPL was extracted from each plasma sample for subsequent quantification. For this purpose, a plasma sample was added into a stoppered tube containing 1 mL mixture of dichloromethane and diethyl ether (4:6, v/v). The resultant solution was shaken on a reciprocating shaker for 30 min at a speed of 100 strokes/min followed by centrifugation at 5000 rpm for 10 minutes. The organic phase was then removed and dried through evaporation under the nitrogen stream. Finally, the residue was reconstituted with 0.2 mL of mobile phase and filtered through a 0.22 μm filter.

The concentration of EPL in each processed sample of plasma was determined with an HPLC system (1200 series, Agilent Tech, Santa Clara, CA) by using a method previously reported (Gide et al., 2012). The chromatographic separation process was accomplished in a Sharpsil-U C18 column (5 μm, 4.6 × 250 mm). The mobile phase was a mixture of acetonitrile and water (50:50, v/v) pumped at a flow rate of 1.0 mL/min. The column temperature was kept at 40 ± 0.5 °C and EPL was quantified at 241 nm using a UV detector system. Data acquisition and peak integration were performed with the Agilent ChemStation software (B.03.01.317, Santa Clara, CA).

#### 2.7.3. Pharmacokinetic parameters

The pharmacokinetics parameters including area under the plasma concentration–time curve to infinite time (AUC_0→∞_), area under the plasma concentration–time curve to last time point (AUC_0→_*_t_*), mean residence time (MRT), maximum plasma EPL concentration (*C*_max_), time to achieve maximum plasma concentration (*T*_max_), plasma half-life (*t*_1/2_), elimination rate constant (*K*_el_), and relative bioavailability (*F*_rel_) were determined by performing non-compartmental analysis using WinNonlin^TM^ software (Version 5.2, Scientific Consulting Inc., Apex, NC). The *F*_rel_ of EPL-NCs compared to EPL powder was calculated by using the following equation:

$$F_{\mathrm{rel}}\;(\%)=\frac{{\mathrm{AUC}}_{0\to t}\text{ of EPL}-\mathrm{NCs}}{{\mathrm{AUC}}_{0\to t}\text{ of EPL powder}}\times 100$$

### 2.8. Acute oral toxicity study of EPL-NCs

Single-dose acute toxicity of EPL-NCs and EPL after oral administration was evaluated in mice following OECD 425 guidelines. Mice were randomly divided into three groups (*n* = 6). The mice in their respective groups were either given normal saline, EPL powder, or EPL-NCs (dispersed in a minimum volume of water) using oral gavage. EPL-NCs and EPL powder were administered in a single dose equivalent to 50 mg/kg of EPL on day 0. Mice in each group were monitored daily for any changes in their body weights, behavioral attributes, physical appearance, a sign of illness, food consumption, or occurrence of toxicology symptoms and mortality for 14 days (Saleem et al., 2016; Sohail et al., 2018).

#### 2.8.1. Determination of hematological and serum biochemistry parameters

On day 14, blood from each mouse was collected from the tail vein to determine hematological and serum biochemistry parameters. For hematological analysis, blood samples in EDTA tubes were analyzed for white blood cells (WBCs count), red blood cells (RBCs) count, platelets count, and hemoglobin (Hb) levels with an automatic hematology analyzer (KX-21, Sysmex Corporation, Kobe, Japan).

For serum biochemistry analysis, serum was separated from the blood samples by centrifugation at 4500 rpm for 15 min and was stored at −20 °C until analyzed. The parameters of renal and liver function tests, the concentration of serum enzymes, proteins, bilirubin, and electrolytes were determined with standard kits using a semi-automated biochemistry analyzer (HumaLyzer 3500, Human Diagnostics, Wiesbaden, Germany).

#### 2.8.2. Organ-to-body weight index

After blood collection on day 14, each mouse was euthanized and vital organs including heart, kidney and liver were carefully removed. The organs were thoroughly washed with the normal saline and the weight of each organ was measured individually. The organ-to-body weight index of mice was calculated by using the following equation (Sarwar et al., 2018):

$$\mathrm{Organ}-\mathrm{to}-\text{body weight index }(\%)=\frac{\text{organ weight }(g)}{\text{body weight }(g)}\times \;100$$

#### 2.8.3. Histopathological examination of organs tissues

Liver, heart, and kidney tissues were preserved by fixing them in 4% neutral buffered paraformaldehyde solution (Zeb et al., 2017b; Yu et al., 2021b). Each organ was then embedded in a paraffin block and sectioned into 0.5 μm thick slices by using a microtome. The prepared sections were mounted on a glass slide, stained with hematoxylin and eosin (H&E) and examined under a light microscope (model CX43, Olympus Corporation, Tokyo, Japan) for morphological changes and signs of toxicity.

### 2.9. Hemolysis assay

The effects of EPL-NCs on the structural integrity of RBCs were evaluated by performing a hemolysis assay on fresh human blood (Yu et al., 2021a). For this purpose, blood (10 mL) was collected from a healthy volunteer in EDTA tubes and centrifuged at 3000×*g* for 10 min to collect the RBCs portion. RBCs were washed with isotonic PBS solution five times for the removal of serum proteins and debris. The purified RBCs were diluted 10-folds with a sterile isotonic PBS solution. Subsequently, 300 µL of the diluted RBCs suspension was filled in a series of different Eppendorf tubes. Sterile isotonic PBS solution (1200 µL) was added to RBCs suspension to serve as a negative control (0% lysis), whereas 1% Triton X-100 solution (1200 µL) was mixed with RBCs suspension to serve as a positive control (100% lysis). Similarly, different concentrations of EPL-NCs and EPL (20, 40, 60, 80, and 100 µg/mL) were mixed with a specified volume of RBCs suspension (300 µL) to check their effect on hemolysis. All these Eppendorf tubes were vortexed and allowed to rest at room temperature for 2 h. Samples were then centrifuged at 13,000×*g* for 10 min, supernatants were collected and absorbances were measured at 541 nm by using a UV–visible spectrophotometer. Finally, hemolysis in each sample was calculated by using the following formula:

$$\mathrm{Hemolysis}\;(\%)=\frac{{\mathrm{Abs}}_{\mathrm{sample}}-{\mathrm{Abs}}_{\text{negative control}}}{{\mathrm{Abs}}_{\text{positive control}}-{\mathrm{Abs}}_{\text{negative control}}}\times \;100$$

### 2.10. Stability studies of EPL-NCs

Short-term storage stability study of optimized EPL-NCs was conducted by following the guidelines of the International Council for Harmonization (ICH). Briefly, EPL-NCs samples were stored in tightly closed glass vials at 40 °C and 75% relative humidity in the stability chamber (Kurakula et al., 2015; Din et al., 2017). EPL-NCs were evaluated on day 0 and 90 for changes in their particle size and dissolution efficiency. In addition, the stability of EPL-NCs was further confirmed by analyzing their DSC thermograms and PXRD pattern at day 0 and 90.

### 2.11. Statistical analysis

All experiments in this study were conducted at least in triplicate and results are reported as mean ± S.D. The statistical analyses were carried out by using GraphPad Prism software (version 9.2.0, San Diego, CA). Student’s *t*-test or one-way ANOVA with post hoc Dunnett’s test was applied to determine the statistical difference among the groups. *p*<.05 was considered as a statistically significant difference among the groups.

## 3. Results and discussion

### 3.1. Design and development of EPL-NCs

NCs are emerged as promising tactic in drug delivery to tune the physiochemical attributes of drugs. Herein, EPL-NCs were designed and developed for enhancing the solubility, dissolution, and oral bioavailability of EPL. Tertiary butyl alcohol was utilized as a solvent for the development of EPL-NCs owing to its pharmaceutical acceptance, low toxicity concerns, and favorable properties for lyophilization (Van Drooge et al., 2004; Mohammady et al., 2020). Mannitol was chosen as a cryoprotectant because of its ability to efficiently crystallize during freeze drying, promote drying, and control crystal growth (Kaialy et al., 2016; Kumar et al., 2017). To reach the optimal amount of cryoprotectant, the concentration of mannitol was kept in the range of 3–15% (Sameti et al., 2003). The independent and dependent variable and the associated levels and constraints are described in Table 1. Since the oral bioavailability of poorly water drugs mainly depends upon the dissolution rate which in turn is affected by the particle size, therefore, particle size and dissolution rate were chosen as key responses for the optimization of a NC formulation.

D-optimal combined mixture design was used to study the effects of independent variables on the dependent variables (NCs size and dissolution efficiency). Optimal mixture design is an efficient screening technique to find out the optimal process and formulation composition. Among the optimal designs, D-optimal design is a favorable tool to determine key variables and evaluate their effects (Jeirani et al., 2012). A total of 15 runs of formulation with different compositions and process variables were generated by the Design-Expert software (Stat-Ease Inc., Minneapolis, MN) using D-optimal mixture design. These 15 formulations were then prepared in triplicate according to the runs generated by software and their mean particle size and dissolution efficiency in 30 min were measured and the results are presented in Table 2. The fraction (percent) of total drug dissolved in 30 min was described as dissolution efficiency of EPL-NCs in our study.

**Table 2.** The experimental runs generated by the optimal design and measured responses for the prepared EPL-NCs.

Run	Factor 1 (*A*+*B*)		Factor 2	Factor 3	Response 1	Response 2
	*A*: solvent (mL)	*B*: antisolvent (mL)	*C*: cryoprotectant concentration (%)	*D*: freezing method	Nanocrystal size (nm)	Dissolution efficiency (%)
1	1	9	3	Rapid	80.1 ± 1.4	70.0 ± 1.3
2	1	9	3	Slow	1076.0 ± 2.7	47.0 ± 0.9
3	1	9	15	Slow	955.0 ± 3.6	55.0 ± 1.1
4	1	9	3	Slow	1316.0 ± 2.9	46.0 ± 1.4
5	1	9	15	Slow	1020.0 ± 3.8	51.0 ± 1.0
6	3	7	15	Rapid	45.5 ± 0.8	95.0 ± 1.6
7	5	5	3	Rapid	54.4 ± 1.1	89.0 ± 2.5
8	5	5	3	Slow	62.5 ± 0.9	75.0 ± 0.7
9	5	5	15	Slow	60.9 ± 0.7	86.0 ± 1.1
10	9	1	15	Rapid	61.3 ± 1.3	77.0 ± 0.8
11	9	1	15	Slow	228.8 ± 2.1	67.0 ± 0.4
12	9	1	15	Slow	349.0 ±1.7	65.0 ± 1.6
13	9	1	15	Rapid	61.6 ± 0.6	78.0 ± 2.2
14	9	1	3	Slow	541.0 ± 1.2	64.0 ± 0.9
15	9	1	3	Slow	635.0 ± 0.9	59.0 ± 0.6

The amount of EPL was 200 mg in all formulations. For the responses, data are presented as mean ± S.D. (*n* = 3).

The results of statistical analysis of the experimental data demonstrate that the fitted models showed significant model *p* values (<.05) and non-significant lack of fit *p* values (>.05) for measured responses of both dependent variables (Table 3). In addition, high *R*^2^ values (close to 1) suggested that the fitted models were effective to predict the responses with high accuracy (Carneiro et al., 2020). Previous studies suggest that best fit model should have a significant *p* value of <.05, non-significant lack of fit *p* value of >.05, acceptable precision value of >4 and difference between adjusted *R*^2^ and predicted *R*^2^ values should be <0.2 (Aksoylu Özbek & Günç Ergönül, 2020). Polynomial equations given in Table 3 describe the fitted models in terms of coded factors built on the base of ANOVA results. These coded equations are useful to identify the relative impact of the factors by comparing their coefficients. The coefficients with *p* value ˂.05 were considered significant. Positive sign in the polynomial equations shows a direct relation of independent variable to response, whereas negative sign represents inverse relation.

**Table 3.** Statistical analysis of the experimental data with ANOVA and the predicted and observed results of the optimized EPL-NCs.

Response	*F*-value	Model *p* value	Lack of fit	Lack of fit *p* value	*R* ^2^	Adjusted *R*^2^	Predicted mean value	Range for mean values at 95% confidence	Obtained value	Error (%)
Nanocrystal size (nm)	151.24	<.0001	1.18	.4053	0.9913	0.9847	45.2	42.94–47.46	46.8 ± 0.5	3.5
Dissolution efficiency (%)	112.46	<.0001	0.516	.6254	0.9912	0.9824	95.8	91.01–100.59	96.0 ± 1.5	0.2

The polynomial equations generated after the statistical analysis are as follow:

Nanocrystal size=+5.23A + 5.68B−5.97AB−0.3330AC−0.7776AD−1.31BD+3.54ABD.

Dissolution efficiency=+69.33A + 61.05B+91.33AB+2.43AC+5.39AD +3.01BC+11.12BD +13.20ABC.

*p* < .05 represents statistically significant difference. For the obtained values, data are presented as mean ± S.D. (*n* = 3).

Error (%)=(obtained value – predicted value)/predicted value × 100.

### 3.2. Effects of mixture and process variables on size of EPL-NCs

The effects of all the independent variables (solvent to antisolvent ratio, cryoprotectant concentration, and freezing method) on the size of EPL-NCs was evaluated. The influence of the corresponding independent factor on response variable was specified by the magnitude of regression coefficients. As our major aim was to estimate the combined effect of three independent variables to find out an optimal composition, the magnitude of linear effect parameters was explicated. The polynomial equations for NC size showed that the mixture components (*A*: solvent, *B*: antisolvent) have major effects on NC size as compared to the process factors (*C*: cryoprotectant concentration, *D*: freezing method). Among the individual mixture components, *B* has a slightly greater effect on NC size as the magnitude of *B*>*A*, whereas the combined effect of these components (*AB*) has a negative impact on NC size. Similarly, the combined effect of mixture component *A* and process factors *C* and *D* have negative influence on the NC size. The positive value of *ABD* with a relatively higher magnitude indicated the synergetic effect of these components.

**Figure 1.**
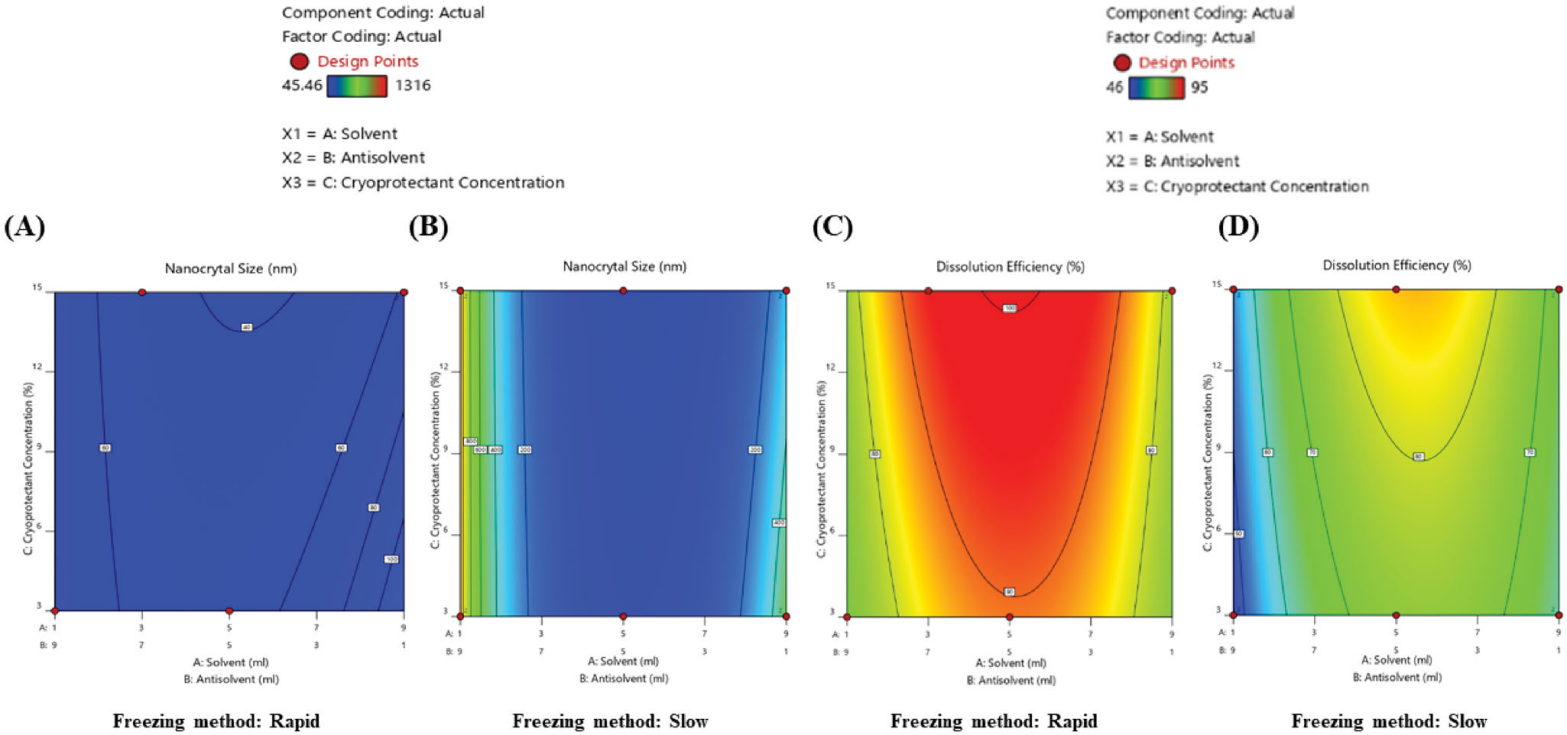
2D contour plots presenting the effects of independent variables on size (A, B) and dissolution efficiency (C, D) of EPL-NCs.

The 2D contour and 3D response surface plots directly show the relationship among the independent variables and their response to changing levels of components (Radfar et al., 2020). The 2D contour (Figure 1(A,B)) and 3D response surface plots (Supplementary Fig. 2A and B) demonstrated that size of EPL-NCs was reduced when solvent to antisolvent ratio was increased from 1:9 to 3:7 (v/v). However, further increase in solvent volume in formulation mixture produced larger EPL-NCs. Previously, it has been reported that highly crystalline solid dispersions of fenofibrate were obtained with a mixture containing higher proportion of organic solvent (6:4, v/v) than water content (De Waard et al., 2008). It could possibly be due to the faster nucleation rate and increased nuclei formation at a lower water content in freeze-drying solution, thereby resulting in lower crystal growth rate and smaller NCs (Mohammady et al., 2020). It is noteworthy to mention that increasing solvent to antisolvent ratio beyond a certain level might change the saturation point and equilibrium between nuclei formation and crystal growth rate, thereby producing larger NCs (Gol et al., 2018; Wu et al., 2019).

The plots further demonstrated that smaller NCs were formed at higher cryoprotectant concentration. In general, fast freezing rate and higher cryoprotectant concentration favor freeze-drying process with the formation of smaller nanoparticles (Lee et al., 2009). In another study, it was found that erlotinib NCs were not stable at lower mannitol concentrations (2–5%) and at least 10% cryoprotectant concentration was required to obtain stable system (Thakkar et al., 2018). The presence of mannitol during lyophilization process attenuates the interactions among NCs and an optimal mannitol concentration is required for successful prevention of aggregate formation. Consequently, a higher mannitol concentration ensures the lowest interaction between EPL-NCs during freeze drying process (Allison et al., 2000; Amis et al., 2020). Our results are in agreement with the previous literature and favor the formation of smaller EPL-NCs at higher mannitol concentration (15%).

After mixing of solvent and antisolvent solutions, the mixtures were frozen either rapidly or slowly before lyophilization. The mixtures were instantaneously placed in −80 °C in rapid freezing method, whereas in slow freezing method the mixtures were slowly cooled to −25 °C at a rate of 0.5 °C/min. As shown in the counter plots and response surface plots, freezing method also influences NCs size. Smaller EPL-NCs were produced with rapid freezing technique compared to those obtained by slow freezing. It is suggested that freezing rate affects the size of interstitial spaces between the solvent crystals. The application of slow freezing rates results in large solvent crystals having large interstitial spaces, and vice versa in case of rapidly freezing the solution. Since crystal formation takes place in freeze-concentrated portion, the final size of drug NCs could be controlled by limiting the interstitial spaces between the solvent crystals (De Waard et al., 2008; Mohammady et al., 2020).

### 3.3. Effects of mixture and process variables on dissolution efficiency of EPL-NCs

From the polynomial equation for dissolution efficiency, it was obvious that all the linear significant model terms of the equation have a positive effect on the dissolution efficiency. Among the mixture components, component *A* has a greater effect on dissolution efficiency than that of component *B*. It was also observed that individual effect of any of the mixture components (*A* or *B*) was higher in combination with process factor *D* compared to their combination with factor *C*. The significant model term *ABC* also has a direct relationship with dissolution efficiency.

The dissolution efficiency of all the prepared EPL-NCs was measured for 30 min and the effects of independent variables on dissolution efficiency was evaluated. The results of statistical analysis by the Design-Expert software (Stat-Ease Inc., Minneapolis, MN) indicated that lower dissolution efficiency was achieved at both the extremes of solvent to antisolvent ratios (1:9 or 9:1), where dissolution efficiency was significantly improved at the intermediate ratios (Figure 1(C,D), and Supplementary Fig. 2C and D). The higher dissolution efficiency of EPL-NCs could be correlated to their relatively smaller sizes at the intermediate ratios of solvent to antisolvent.

The concentration of cryoprotectant also affected the dissolution efficiency of EPL-NCs. As shown in the results, dissolution efficiency was higher for EPL-NCs prepared with the upper limit of mannitol (15%) than those with lower mannitol (3%) concentration. The enhanced dissolution efficiency with higher mannitol concentration is due to improved porosity and wettability of EPL-NCs thereby achieving efficient penetration of dissolution medium and faster solubilization (Jaipal et al., 2015; Kaialy et al., 2016). Furthermore, smaller NCs were obtained with upper level of cryoprotectant concentration (15%) which could have contributed in achieving better dissolution efficiency due to increased surface area.

EPL-NCs demonstrated better dissolution efficiency when prepared with rapid freezing method as compared to those obtained by slow freezing. The rate of freezing affected the size of EPL-NCs, therefore it was assumed that rapid freezing enhanced their dissolution efficiency owing to reduced NCs size. In a previous study, SEM analysis also confirmed the formation of coarse particles when solutions were frozen slowly (De Waard et al., 2008).

### 3.4. Optimization of EPL-NCs

The numerical optimization of Design-Expert with desirability function was used to attain the final optimized formulation for further studies. The goal for all mixture and process factors was set to in-range with an importance level of 3, whereas the goals for NC size and dissolution efficiency were set to minimize and maximize, respectively, at an importance level of 5. The software generated a total of 100 solutions, out of which a solution was selected with desirability 1, and predicted NCs size of 45.2 nm, and predicted dissolution efficiency of 95.8%. The counter plots of desirability and predicted size and dissolution efficiency of the optimal EPL-NCs formulation are presented in Supplementary Fig. 3. Based on the software analysis, composition and process variables predicted for the optimized EPL-NCs formulation include solvent to antisolvent ratio of 4.5:5.5 (v/v), cryoprotectant concentration of 9% with rapid freezing method to get the desired outcomes.

The optimized EPL-NCs formulation with the above-mentioned predicted mixture and process variables was prepared in triplicate and their size and dissolution efficiency were measured. The obtained results of EPL-NCs indicated average size of 46.8 ± 0.5 nm and dissolution efficiency of 96.0 ± 1.5% at 30 min (Table 3). The accuracy was the model checked by performing error analysis by computing the difference between observed and predicted response values. The results obtained from experiments were close enough to the predicted values. In addition, NC size and dissolution efficiency showed less than 5% error which validated the Design-Expert model (Visetvichaporn et al., 2020). Overall, our results indicated successful optimization of EPL-NCs formulation with D-optimal mixture design approach.

### 3.5. Physicochemical properties of EPL-NCs

**Figure 2.**
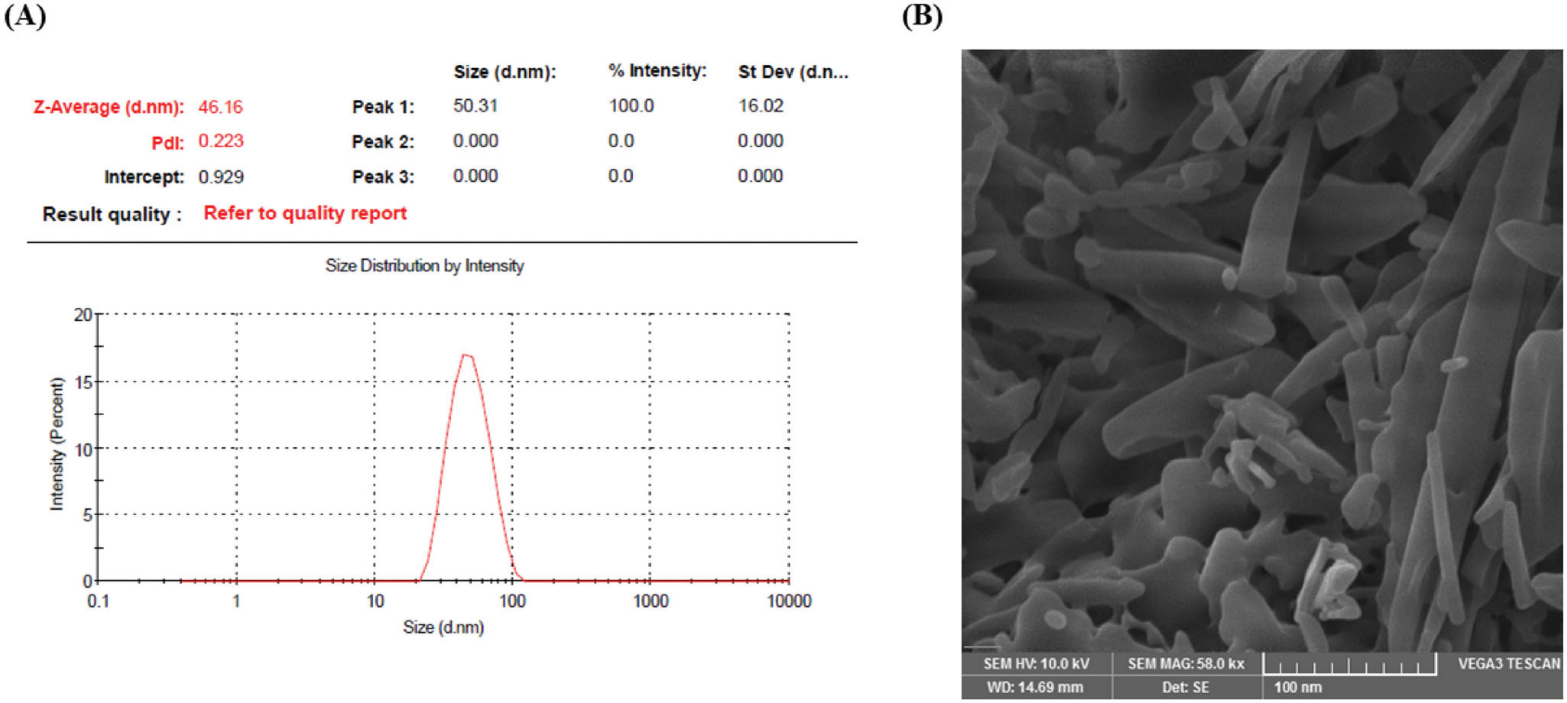
Particle size distribution curve (A) and scanning electron microscopic image (B) of the optimized EPL-NCs.

The physiochemical properties of nano-systems are important factors to determine *in vitro* and *in vivo* performance such as solubility, dissolution, oral absorption, and their ability to reach a particular site. Therefore, the optimized EPL-NCs were subjected to further physicochemical characterization. The particle size analysis by dynamic light scattering technique showed that EPL-NCs possessed a mean diameter of 46.8 ± 0.5 nm with a PDI value of 0.31 ± 0.07. Moreover, particle size distribution curve of EPL-NCs was unimodal in shape (Figure 2(A)) indicating their size uniformity. Formulations having PDI values of up to 0.3 combined with unimodal curves are generally considered as homogenously distributed nanoparticle systems (Khan et al., 2020). The shape and morphological features of the optimized EPL-NCs were analyzed by SEM analysis and the resultant image is presented in Figure 2(B). The SEM result confirmed the formation of elongated parallelepiped shaped crystals with smooth surface morphology. Overall, the results of DLS and SEM are in good agreement to confirm the formation of NCs in nanometric size range and their crystal-like surface morphology.

### 3.6. FTIR, DSC, and PXRD analyses

**Figure 3.**
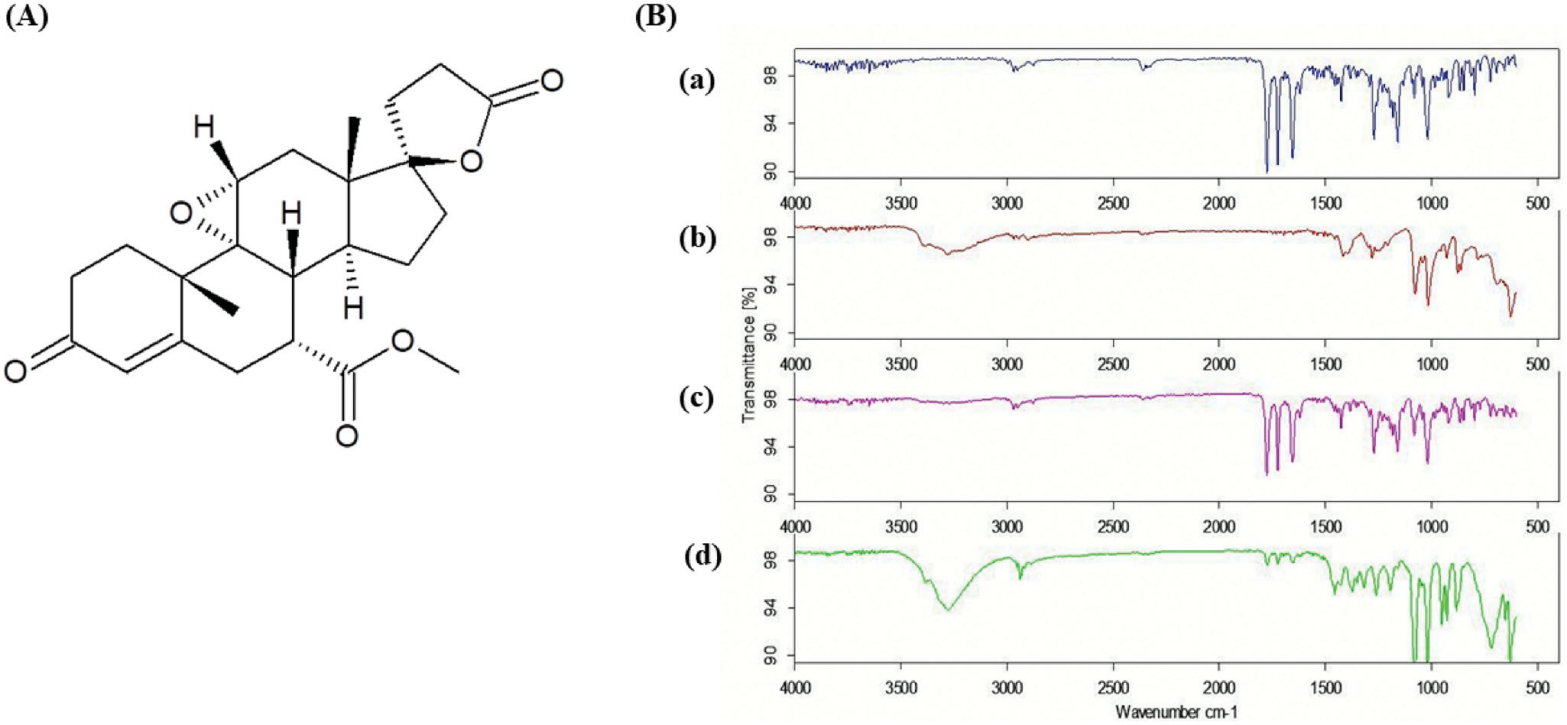
Chemical structure of EPL (A) and FTIR spectra (B) of EPL (a), mannitol (b), physical mixture (c), and EPL-NCs (d).

The molecular interactions of EPL within EPL-NCs were studied by FTIR analysis. The chemical structure of EPL is presented in Figure 3(A), whereas FTIR spectra are shown in Figure 3(B). The spectra of EPL revealed the characteristic absorption bands for main functional groups at 2954.33 cm^−1^ for C–H stretching, 1773.78 cm^−1^ for anhydride O–C=O stretching, 1723.59 cm^−1^ for C=O ester stretching, and 1652.36 cm^−1^ for C=O stretching. The retention of distinct EPL peaks in physical mixture as well as EPL-NCs and appearance of additional peaks for mannitol in EPL-NCs confirmed the compatibility of formulation ingredients and successful formation of NCs without any chemical change (Thakkar et al., 2018; Khames, 2019).

**Figure 4.**
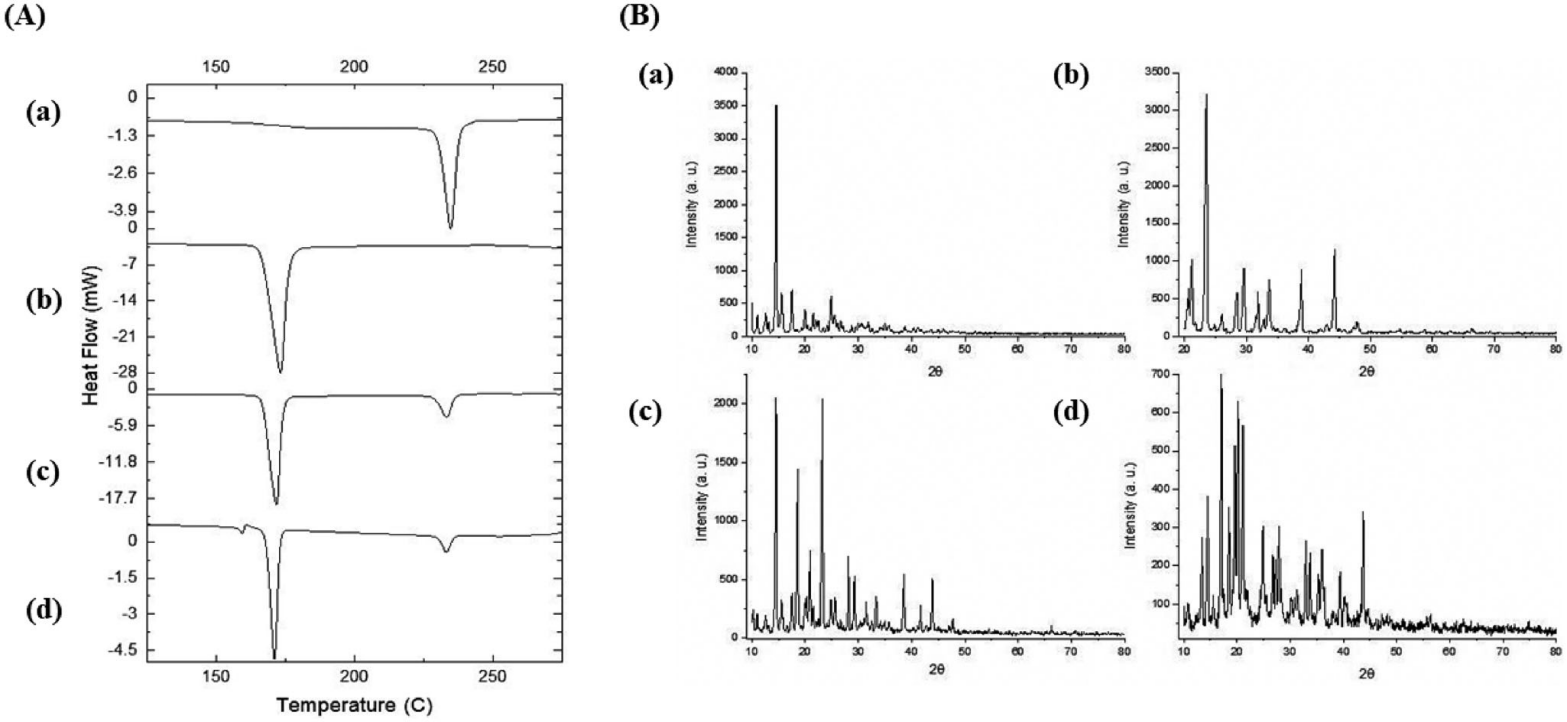
DSC thermograms (A) and PXRD patterns (B) of EPL (a), mannitol (b), physical mixture (c), and EPL-NCs (d).

Thermal analysis of EPL-NCs was performed with DSC to examine changes in crystalline properties of EPL and its compatibility with formulation ingredients (Figure 4(A)). DSC thermograms of EPL and mannitol displayed sharp endothermic peaks at 235 °C and 173 °C, which correspond to their respective melting points. The melting peaks of EPL and mannitol remained unchanged in thermal scans of physical mixture and EPL-NCs. These results suggest the crystallographic purity of EPL-NCs and existence of compatibility among the formulation ingredients. It is noteworthy to mention that a slight depression in the melting point of EPL was observed in the thermogram of EPL-NCs which could be attributed to its good miscibility with mannitol and reduced particle size (Shete et al., 2015; Thakkar et al., 2018).

The crystalline behavior of EPL-NCs was further confirmed with PXRD and the obtained diffractograms are shown in Figure 4(B). The PXRD pattern of EPL revealed its sharp crystalline peaks at 2*θ* values of 10°, 14.55°, 15.55°, 17.5°, and 24.85°. Similarly, distinct peaks were also observed for mannitol indicating their crystalline nature. All the characteristics peaks of EPL and mannitol were retained in physical mixture and EPL-NCs. A slight decline in the peaks height was observed which could be correlated with changes in crystal size and crystalline habit of EPL in EPL-NCs (Koradia et al., 2018). Despite of these minor change, EPL-NCs powder is still in highly crystalline form without a significant amorphization during processing. Taken together, DSC and PXRD results suggest the successful formation of EPL-NCs by controlled crystallization technique.

### 3.7. Solubility and dissolution profile of EPL-NCs

**Figure 5.**
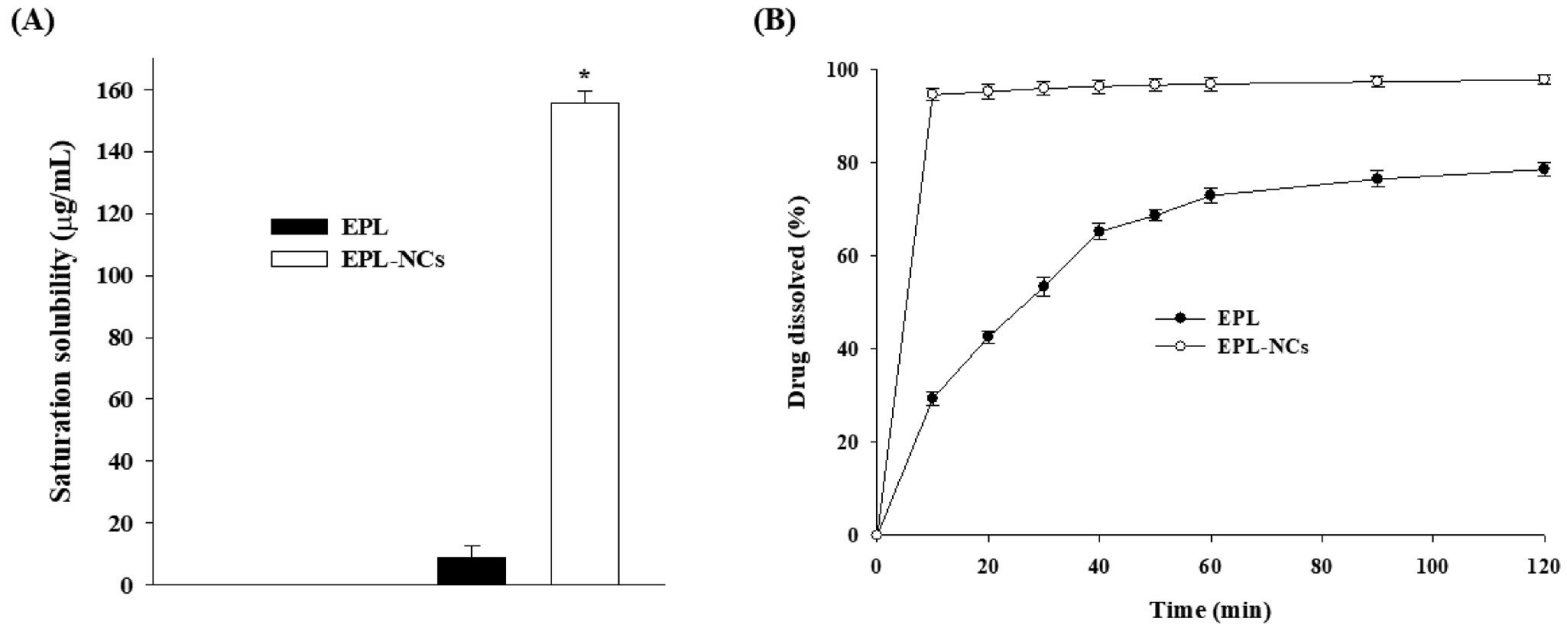
Saturation solubility (A) and dissolution profile (B) of the optimized EPL-NCs. Data are presented as mean ± S.D. (*n* = 3). *p<.001 vs. EPL powder.

The saturation solubility and dissolution of pure EPL powder and EPL-NCs were determined in 0.1 N HCl solution to stimulate the gastric fluid. Since 37 °C represents the relevant physiological temperature; therefore, saturation solubility of EPL-NCs was determined at body temperature rather than the room temperature (Plöger et al., 2018). Our results demonstrated (Figure 5(A)) that saturation solution of EPL-NCs (155.85 ± 3.91 µg/mL) was significantly increased as compared to pure EPL powder (8.96 µg/mL). It turns out to be ∼17-folds increase in the saturation solubility of EPL after formulating in NCs. The increase in saturation solubility of EPL-NCs could be explained on the basis of increased surface area that changes their physiochemical properties. The effect of increased surface area on saturation solubility is significantly important when particle size is reduced below 1000 nm. This phenomenon could be described according to Kelvin’s equation which correlates dissolution pressure with the curvature of solid particles in liquid medium. As particle curvature is increased by decreasing particle size, the dissolution pressure is increased thereby equilibrium is shifted toward solubility and dissolution (Buckton & Beezer, 1992).

The dissolution of EPL-NCs was measured for 120 min and dissolution profile is presented in Figure 5(B). EPL-NCs demonstrated substantially higher dissolution rate compared to that of pure EPL powder as ∼95% of drug was dissolved from EPL-NCs in just 10 min. In contrary, only 29% of EPL powder was dissolved in 10 min that reached up to 53% and 78% in 30 and 120 min, respectively. The enhanced dissolution rate (velocity) of EPL-NCs could be explained on the basis of Noyes–Whitney’s equation which correlates dissolution rate with saturation solubility, surface area, and diffusional distance of the dissolving particles (Junyaprasert & Morakul, 2015). EPL-NCs with reduced particle size offer greater surface area, higher saturation solubility which in turn increases the concentration gradient, and reduced diffusional distance. The combination of these factors increased the dissolution rate of EPL-NCs according to the Noyes–Whitney’s equation.

### 3.8. *In vivo* pharmacokinetics study

**Figure 6.**
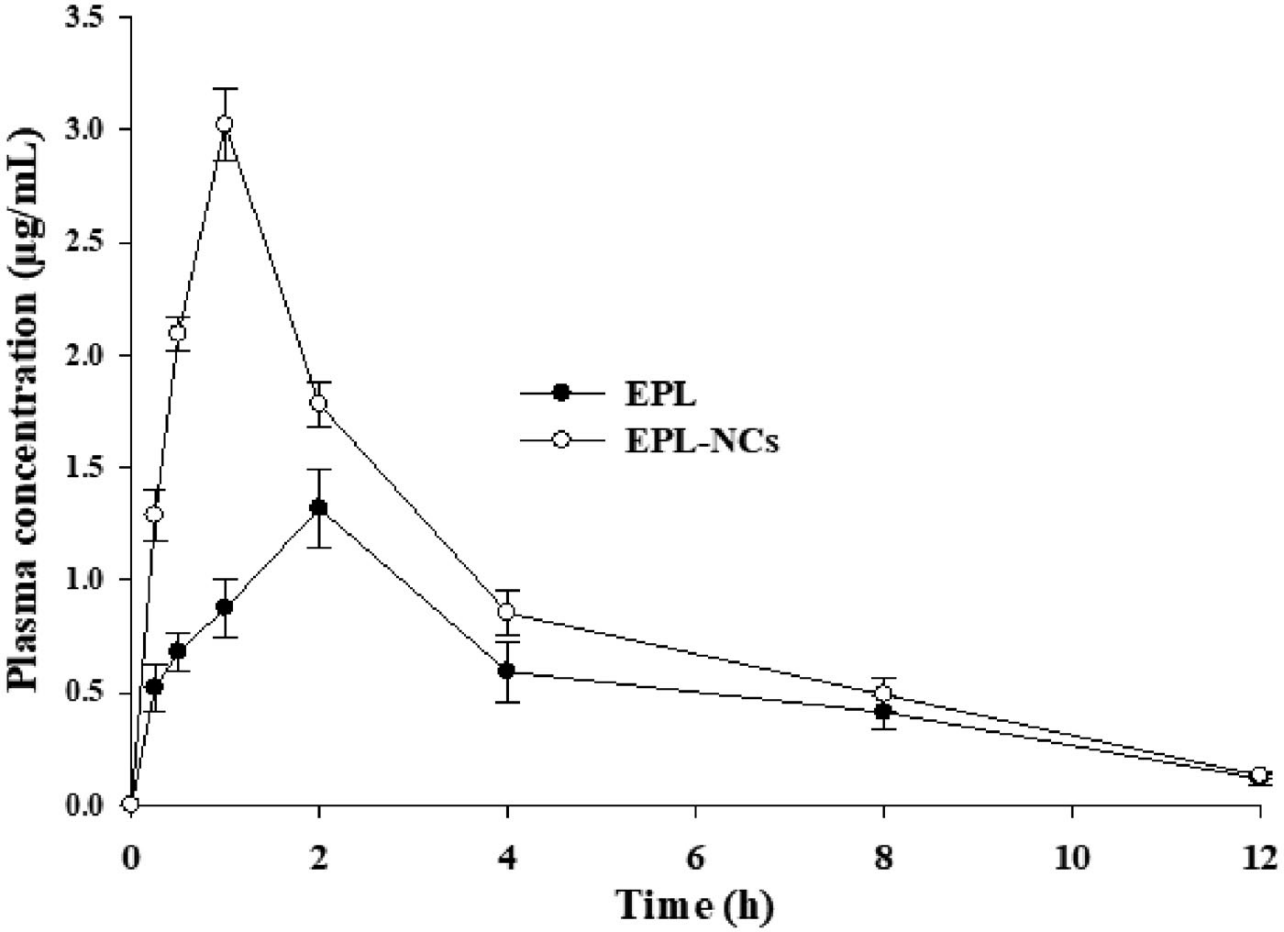
Average plasma level vs. time curve after oral administration of EPL-NCs and EPL powder to rats at a dose equivalent to 10 mg/kg of EPL. Data are expressed as mean ± S.D. (*n* = 3).

The comparison of plasma concentration vs. time curves of EPL-NCs and EPL dispersion after oral administration to rats is shown in Figure 6. The plasma profiles indicated that EPL-NCs achieved higher drug concentration in plasma at every time point compared to those of EPL dispersion in water. The pharmacokinetic parameters calculated from plasma drug concentration vs. time data are given in Table 4. It was observed that the key parameters to describe rate and extent of oral absorption such as AUC_0→_*_t_*, AUC_0→∞_, and *C*_max_ were significantly higher for EPL-NCs as compared to pure EPL powder. In addition, time to reach maximum drug concentration in plasma was substantially reduced for EPL-NCs demonstrating their rapid absorption from the GI tract. The *F*_rel_ of EPL-NCs was about 1.5 times higher when compared to EPL powder. Collectively, these results indicated better oral absorption and subsequent higher bioavailability of EPL-NCs, which could be ascribed to their reduced particle size and increased surface area. The findings of *in vivo* pharmacokinetics study are well supported by the *in vitro* saturation solubility and dissolution data.

**Table 4.** Non-compartmental pharmacokinetic parameters after oral administration of EPL-NCs and EPL powder to rats at a dose equivalent to 10 mg/kg of EPL.

Parameters	EPL	EPL-NCs
AUC_0→_*_t_* (μg·h/mL)	6.66 ± 0.93	10.50 ± 0.42*
AUC_0→∞_ (μg·h/mL)	7.19 ± 1.03	10.96 ± 0.43*
MRT (h)	5.10 ± 0.10	3.90 ± 0.04
*C*_max_ (µg/mL)	1.31 ± 0.14	2.97 ± 0.09*
*T*_max_ (h)	2.0 ± 0.00	1.00 ± 0.00*
*t*_1/2_ (h)	3.12 ± 0.06	2.60 ± 0.03
*K*_el_ (h^–1^)	0.22 ± 0.003	0.27 ± 0.003
*F*_rel_ (%)	–	151.0 ± 37.1

Data are expressed as mean ± S.D. (*n* = 3).

* *p* ˂ .01 vs. EPL.

Oral administration of poorly water soluble drugs is challenging due to their inherent solubility and dissolution problems and drug NCs possess prominent features to solve these problems. NCs have been reported to augment oral absorption and bioavailability of drugs by different mechanisms such as improvement in solubility and dissolution profiles and increasing adhesion to the GI tract (Tian et al., 2021). Small size of drug NCs not only facilitates their rapid dissolution in GI fluid but also increases concentration gradient (due to high saturation solubility) across the GI lumen thereby promoting passive diffusion for absorption (Junyaprasert & Morakul, 2015). In addition, small size and large surface area of NCs promote their adhesion to GI mucosa via electrostatic and van der Waals forces thereby increasing their contact time for efficient oral absorption (Tian et al., 2021). These contributing factors result in better plasma profiles of drug NCs such as increased AUC and *C*_max_, and reduced *T*_max_ as illustrated by the pharmacokinetics parameters of EPL-NCs in this study.

### 3.9. Acute oral toxicity profile of EPL-NCs

With rapid growth of nanotechnology in recent years, a new concept of ‘nanotoxicology’ has emerged due to the capability of nanoparticles to adversely interact with different biological systems and produce toxic responses (Oberdörster et al., 2007). For these reasons, acute toxicity studies on the developed nanosystems are extremely important to evaluate their potential as drug delivery carriers. These studies provide valuable information on the safety profile and potential risk of toxicity on vital organs in small animals (Bulcão et al., 2013; Radwan et al., 2017). The optimized EPL-NCs were therefore evaluated for acute toxicity after oral administration to Swiss albino mice. For this purpose, higher dose of EPL-NCs were utilized (equivalent to 50 mg/kg of EPL) compared to those used for *in vivo* pharmacokinetics study (10 mg/kg). It has been previously reported that the acute toxicity studies on nanosystems are conducted at a dose higher (5–10 times) than those used for therapeutic purposes in order to assess their safety (Bulcão et al., 2013). The animals were monitored for 14 days for any possible toxic effects of EPL-NCs after oral administration. During this period, no mortality or visual signs of toxicity such as changes in behavior, physical appearance, food consumption, and appearance of clinical symptoms were observed. The body weights changes of all animals were measured and results are presented in Supplementary Table 1. EPL-NCs administered mice did not show any significant changes in their body weights compared to EPL and normal groups. The body weights of all groups were slightly increased compared to their weights at day 0, which is due to food consumption for two weeks.

The hematological parameters provide an insight into a possible tissue injury by the tested formulation or compound (Dandekar et al., 2010). The effects of EPL-NCs on hematological parameters are shown in Supplementary Table 2. The results showed that EPL-NCs did not alter any of the parameter of complete blood count compared to normal and EPL group, which indicate their compatibility with blood cells and the absence of injury in the tissues. Further, serum biochemistry was analyzed to check liver and kidney functions and electrolyte imbalance (Supplementary Table 2). Liver and kidney are the main organs responsible for metabolism and excretion, respectively, and therefore are most likely to be affected. EPL-NCs did not significantly affect any of the parameters used to describe liver functions such as serum albumin, bilirubin, alkaline phosphatase (ALP), alanine aminotransferase (ALT), and aspartate aminotransferase (AST) compared to normal and EPL groups. In general, high levels of AST, ALT, and ALP are detected in serum if the cellular integrity of liver cells are compromised (Giannini et al., 2005). Similarly, EPL-NCs did not produce any significant effect on urea and creatinine levels, signifying intactness of the kidney functions. Serum electrolytes, glucose, and cholesterol were in normal ranges for all groups. These results demonstrated that EPL-NCs were safe for blood cells, liver, and kidney functions.

**Figure 7.**
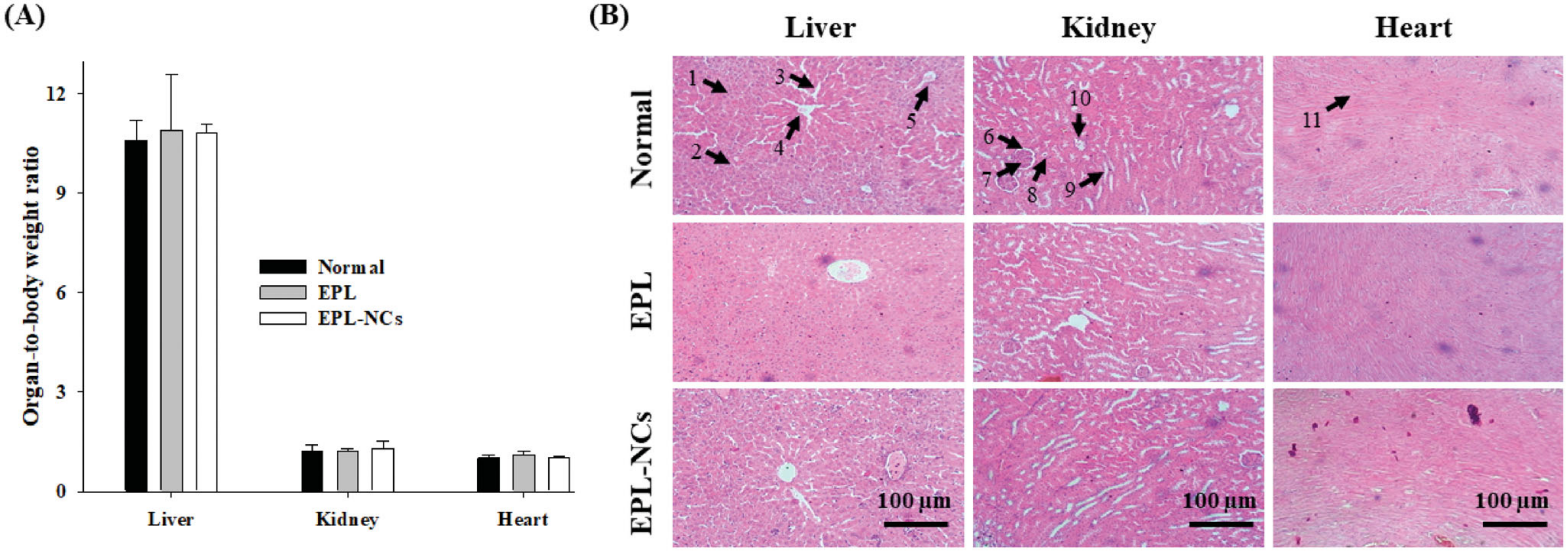
Organ-to-body weight index (A) and histological microphotographs of liver, kidney and heart tissues of mice (B) at day 14 after treatment with EPL-NCs. In histological micrographs, (1) hepatocytes, (2) hepatic artery, (3) sinusoids, (4) central vein, (5) bile duct, (6) bowman capsule, (7) glomerulus, (8) renal tubule, (9) proximal tubule, (10) distal tubule, and (11) myocardial fibers. Organ-to-body weight indices are presented as mean ± S.D. (*n* = 6).

The measurement of organ weight and its ratio with today body weight (organ-to-body weight index) is highly recommended in toxicological investigation of drugs and pharmaceutical formulations (Sellers et al., 2007). Changes in organ-to-body weights are linked with the toxic effects of test compound. EPL-NCs did not produce any significant change in organ-to-body indices of the vital organs including liver, kidney, and heart (Figure 7(A)). The organ-to-body indices of EPL-NCs group were comparable to those of normal and EPL administered mice. Histopathological examination was performed to further investigate the effects of EPL-NCs on structural integrity of heart, kidney, and liver tissues. The histological micrographs demonstrated that EPL-NCs did not affect the cellular morphology of these tissues as no pathological alterations were observed when compared to normal and EPL groups (Figure 7(B)). Normal cellular architecture cardiac myofibrils and myocytes were observed without any gross evidence of damage, necrosis, or infiltration in the heart tissues. Similarly, normal glomerular structures were observed in kidney tissues and hepatocytes displayed normal morphology in liver tissues. The findings of histopathological examinations augment the results from serum biochemistry and organ-to-body indices. Taken the results of acute toxicity together, EPL-NCs are well tolerated without producing any toxic effect after oral administration to mice.

### 3.10. Effects of EPL-NCs on blood hemolysis

**Figure 8.**
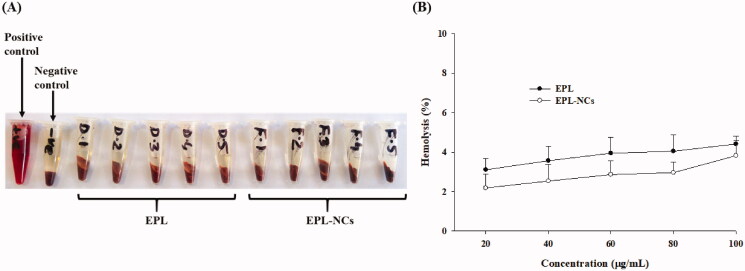
Blood hemolysis by the optimized EPL-NCs indicated with real-time photographs of RBCs samples (A) and quantified with spectroscopic analysis of hemoglobin at 541 nm (B). The RBCs were mixed with Triton X-100 solution (positive control), isotonic PBS (negative control), 20, 40, 60, 80, and 100 µg/mL of EPL (D-1 to D-5) and 20, 40, 60, 80, and 100 µg/mL of EPL-NCs (F-1 to F-5), respectively. For the quantification of hemolysis (%), data are presented as mean ± S.D. (*n* = 3).

Hemocompatibility is very important for biomaterials as they may damage the integrity of erythrocytes in the human body. Nanoparticle systems eventually come in contact with blood cells once they enter the systemic circulation. Small particle size with large surface area render them capable of interacting and disrupting blood cells. Therefore, it is crucial to determine the hemolytic potential of the developed nanosystems to make them acceptable in clinical applications (de la Harpe et al., 2019). In our study, the hemolytic potential of EPL-NCs was evaluated in a concentration range of 20–100 µg/mL. The real-time photographs of EPL-NCs group did not show any substantial hemolysis compared to negative control with 0% lysis (Figure 8(A)). In contrary, 100% lysis of RBCs was observed in samples treated with 1% Triton X-100 solution. Furthermore, quantitative analysis showed that EPL-NCs caused only 3.8 ± 0.8% hemolysis even at a maximum concentration of 100 µg/mL, whereas 2.2 ± 0.7% hemolysis at 20 µg/mL (Figure 8(B)). These findings suggest that EPL-NCs were quite compatible with RBCs causing negligible effects on their integrity. It is worthy to mention that critical safe hemolytic range for biomaterials is 5% as mentioned in ISO/TR 7406 (Sanoj Rejinold et al., 2011). Since the hemolysis ratio caused by EPL-NCs lies within the critical range, EPL-NCs can be considered safe for erythrocytes integrity.

### 3.11. Storage stability of EPL-NCs

**Figure 9.**
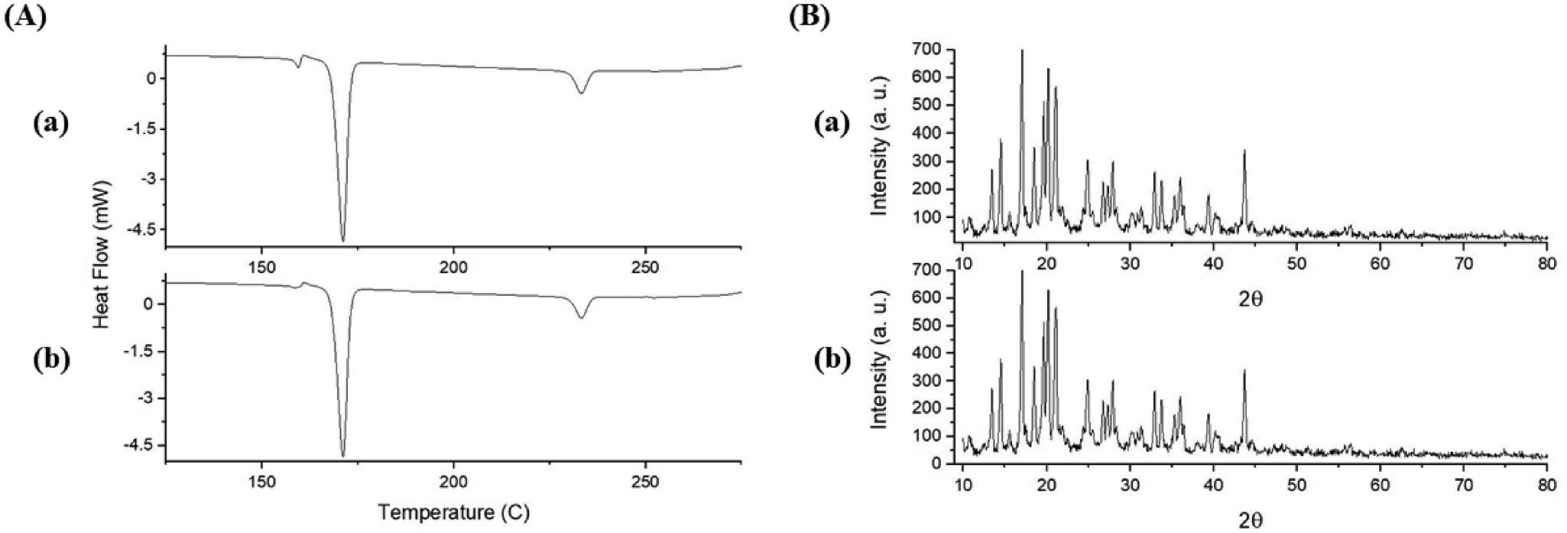
DSC thermograms (A) and PXRD patterns (B) of EPL-NCs at day 0 (a) and after 90 days (b) of storage stability period.

The storage stability of the optimized EPL-NCs was evaluated to monitor their physicochemical properties upon storage. The data of changes in particle size and dissolution efficiency after 90 days of storage at 40 °C are presented in Table 5. No substantial changes were observed in particle size and dissolution efficiency. Furthermore, the crystalline behavior of EPL-NCs was evaluated by DSC and PXRD after 90 days of storage and were compared to the results obtained on day 0 (Figure 9(A,B)). The appearance of characteristics peaks at their original positions suggested that EPL-NCs were stable for at least 90 days as they retained thermal and crystalline behavior upon storage. Based on these findings, it is expected that EPL-NCs would retain *in vitro* properties and *in vivo* performance.

**Table 5.** Storage stability of optimized EPL-NCs at 40 °C for 90 days.

	Day 0	Day 90	Change (%)
Particle size (nm)	46.8 ± 0.5	49.9 ± 2.3	+6.62
Dissolution efficiency (%)	96.0 ± 1.5	93.5 ± 1.0	–2.60

Data are presented as mean ± S.D. (*n* = 3).

## 4. Conclusions

EPL-NCs were successfully optimized by D-optimal design and formulated by using controlled crystallization technique during freeze-drying. The optimized EPL-NCs showed particle size in nanometer range with very high saturation solution and dissolution efficiency of 96%. EPL-NCs improved the oral bioavailability compared to EPL powder. Furthermore, EPL-NCs were safe to vital organs and biocompatible with blood. Hence, EPL-NCs prepared by this technique could be utilized for potential development of oral dosage form with commercial scalability. In conclusion, controlled crystallization during freeze-drying might be a promising technique to overcome the drawbacks of bottom-up approaches and could be used to develop NCs of other water insoluble drugs.

## Supplementary Material

Supplemental Material
