# Supplementary material for: Eplerenone nanocrystals engineered by controlled crystallization for enhanced oral bioavailability
Source: Drug Deliv. 2021 Nov 29;28(1):2510–24. doi: 10.1080/10717544.2021.2008051 (PMC8635601; doi:10.1080/10717544.2021.2008051)
Supplement: Supplemental Material [file IDRD_A_2008051_SM2378.docx]

**Supplementary material**

**
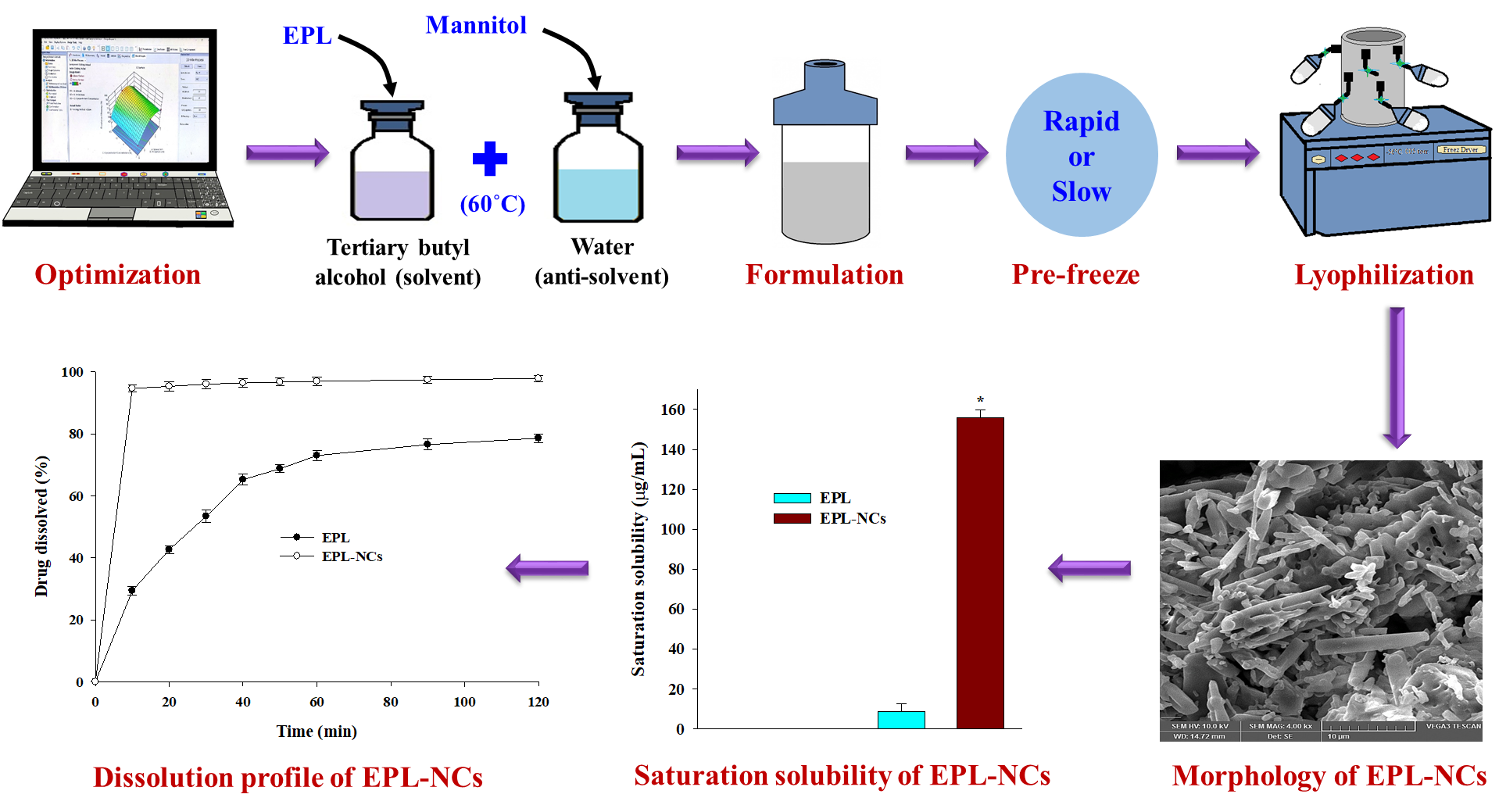
**

**Suppl. Fig. 1.** Graphical abstract representing the preparation, optimization and characterization of EPL-NCs.

**(B)**

**(A)**

**
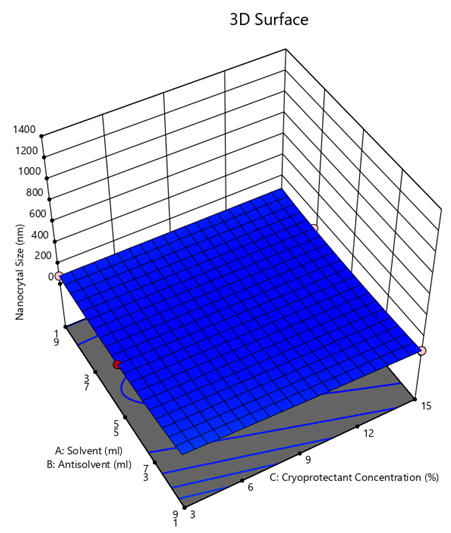

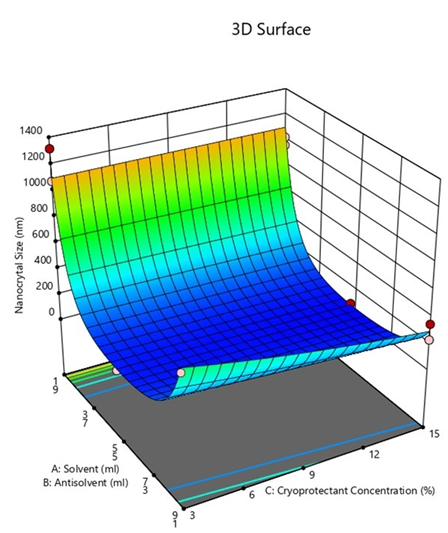
**

**Freezing method: Slow**

**Freezing method: Rapid**

**(D)**

**(C)**

**
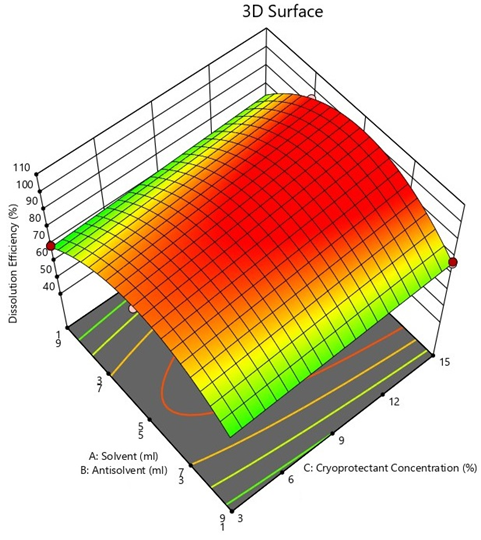

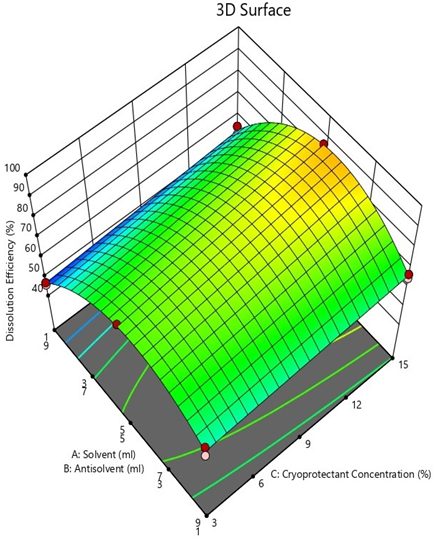
**

**Freezing method: Slow**

**Freezing method: Rapid**

**Suppl. Fig. 2.** 3D response surface plots presenting the effects of independent variables on size (A, B) and dissolution efficiency (C, D) of EPL-NCs.

**(B)**

**(A)**


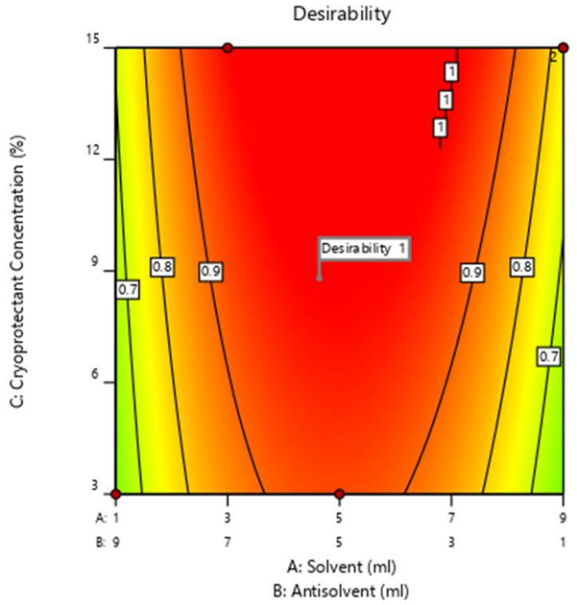

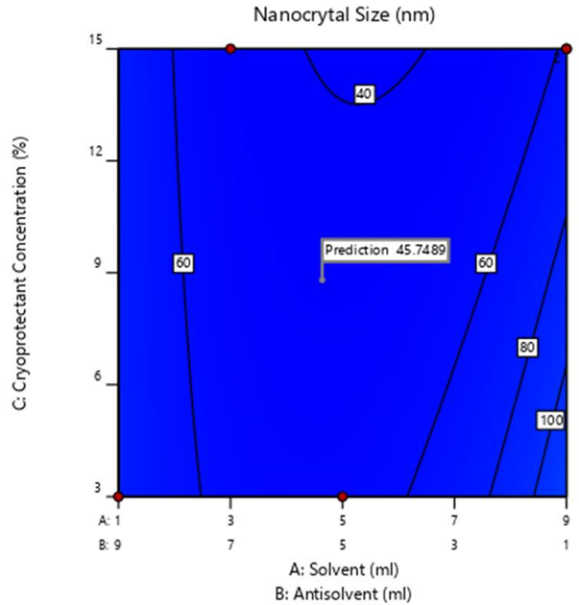


**(C)**

**
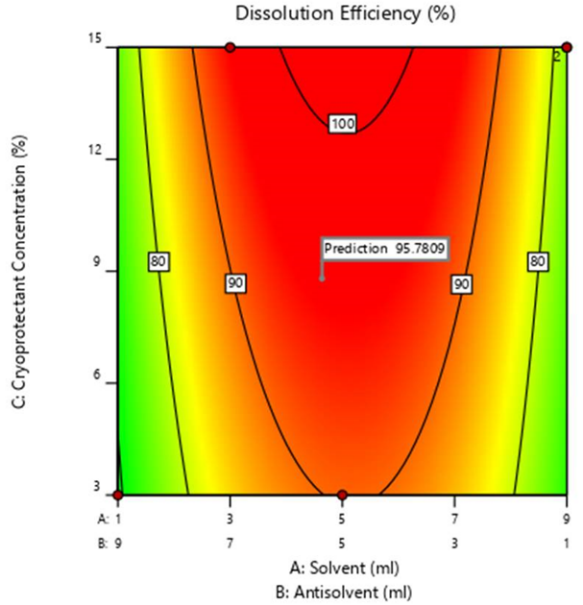
**

**Suppl. Fig. 3.** Contour plots of desirability (A), and the predicted size (B) and dissolution efficiency (C) of the optimal EPL-NCs formulation.

**Suppl. Table 1**

Body weight changes of mice in acute toxicity studies after oral administration of EPL-NCs and EPL powder at a dose equivalent to 50 mg/kg of EPL.

| Groups | Body weight (g) | |
| --- | --- | --- |
|  | **Day 0** | **Day 14** |
| Normal | 31.0 ± 1.6 | 31.7 ± 1.6 |
| EPL | 30.7 ± 0.9 | 31.9 ± 1.0 |
| EPL-NCs | 30.1 ± 0.7 | 30.8 ± 0.6 |

Body weights are presented as mean ± S.D. (n = 6).

**Suppl. Table 2**

Hematological and serum biochemistry analysis in acute toxicity studies after oral administration of EPL-NCs and EPL powder to mice at a dose equivalent to 50 mg/kg of EPL.

| **Hematological analysis** | **Parameters** | **Normal** | **EPL** | **EPL-NCs** |
| --- | --- | --- | --- | --- |
|  | WBCs count (×10^9^/L) | 15.4 ± 2.2 | 14.9 ± 2.8 | 15.7 ± 2.9 |
|  | RBCs count (×10^12^/L) | 8.3 ± 2.1 | 8.6 ± 3.2 | 8.2 ± 2.5 |
|  | Platelets count (×10^9^/L) | 735.6 ± 80.8 | 729.7 ± 71.8 | 732.2 ± 72.9 |
|  | Hematocrit (%) | 40.8 ± 2.4 | 41.9 ± 3.8 | 41.5 ± 1.7 |
|  | Hb (g/dL) | 13.9 ± 2.5 | 13.8 ± 3.3 | 14.1 ± 4.5 |
|  | Mean cell volume (fL) | 49.9 ± 4.9 | 50.2 ± 3.7 | 49.7 ± 3.7 |
|  | Mean cell hemoglobin (pg) | 14.7 ± 2.3 | 15.2 ± 3.2 | 15.5 ± 3.6 |
|  | Packed cell volume (%) | 47.7 ± 4.4 | 48.3 ± 7.3 | 49.5 ± 3.9 |
| **Serum biochemistry** | Albumin (g/dL) | 2.5 ± 0.3 | 2.5 ± 0.3 | 2.4 ± 0.2 |
|  | Total bilirubin (mg/dL) | 0.2 ± 0.04 | 0.2 ± 0.03 | 0.2 ± 0.02 |
|  | ALP (IU/L) | 96.1 ± 17.3 | 95.2 ± 17.3 | 94.8 ± 31.2 |
|  | ALT (IU/L) | 60.3 ± 5.4 | 58.3 ± 3.3 | 57.9 ± 2.7 |
|  | AST (IU/L) | 98.2 ± 33.4 | 97.1 ± 21.8 | 95.3 ± 12.6 |
|  | Urea (mg/dL) | 22.7 ± 3.3 | 22.3 ± 4.2 | 21.9 ± 2.4 |
|  | Creatinine (mg/dL) | 0.4 ± 0.04 | 0.4 ± 0.03 | 0.4 ± 0.03 |
|  | Sodium (mEq/L) | 155.5 ± 3.3 | 152.6 ± 1.6 | 150.2 ± 3.6 |
|  | Potassium (mEq/L) | 5.3 ± 0.5 | 5.9 ± 0.9 | 6.7 ± 0.7 |
|  | Calcium (mg/dL) | 9.6 ± 0.9 | 9.2 ± 1.4 | 9.4 ± 0.9 |
|  | Phosphorus (mg/dL) | 6.9 ± 0.8 | 6.6 ± 0.9 | 6.4 ± 1.5 |
|  | Glucose (mg/dL) | 211.2 ± 14.6 | 209.2 ± 14.2 | 202.5 ± 12.8 |
|  | Cholesterol (mg/dL) | 148.4 ± 16.3 | 153.5 ± 19.9 | 155.7 ± 16.4 |
|  | Total protein (g/dL) | 5.3 ± 0.9 | 5.4 ± 0.5 | 5.5 ± 0.7 |

Data are presented as mean ± S.D. (n = 3).
